# Novel nerve-sparing robot-assisted radical prostatectomy with endopelvic fascia preservation and long-term outcomes for a single surgeon

**DOI:** 10.1038/s41598-024-51598-3

**Published:** 2024-01-09

**Authors:** Masafumi Maruo, Yusuke Goto, Kanetaka Miyazaki, Atsushi Inoue, Koichiro Kurokawa, Akiko Enomoto, Satoki Tanaka, Sota Katsura, Sho Sugawara, Miki Fuse, Kazuto Chiba, Yusuke Imamura, Shinichi Sakamoto, Maki Nagata, Tomohiko Ichikawa

**Affiliations:** 1 Department of Urology, Chiba University Graduate School of Medicine, 1-8-1, Inohana, Chiba Japan; 2 Department of Urology, Yokohama Rosai Hospital, Kanagawa, Japan

**Keywords:** Prostate, Surgical oncology

## Abstract

Although novel techniques for avoiding incontinence during robot-assisted radical prostatectomy have been developed, long-term oncological outcomes are unknown. The objective of this study was to determine the long-term oncological outcomes and functional outcomes of novel nerve-sparing robot-assisted radical prostatectomy with endopelvic fascia preservation for a single surgeon. Data from 100 patients who underwent structure-preserving prostatectomies performed by a single surgeon were retrospectively analyzed. The median console time was 123 min. Bilateral nerve-sparing was performed in 43% of patients underwent, and 57% underwent unilateral nerve-sparing surgery. Most patients (96%) reached complete pad-zero urinary continence by one year after surgery. Satisfactory erectile function was achieved in 97% of patients who underwent bilateral nerve-sparing surgery, and 80% of patients who underwent unilateral nerve-sparing surgery. The surgical margin was positive for 25% of patients, and the biochemical recurrence-free rate at 5 years was 77%. The cancer-specific survival rate was 100% during the median follow-up period of 4.5 years. Clavien-Dindo grade III complications occurred in 1% of cases. The outcomes for novel nerve-sparing robot-assisted radical prostatectomy with endopelvic fascia preservation were similar to previously reported oncological outcomes, with satisfactory functional outcomes. This operative method may be useful for patients who are eligible for nerve-sparing surgery.

## Introduction

Robot-assisted prostatectomy (RARP) provides a magnified and detailed view of anatomical structures around the prostate, facilitating finer surgical techniques. Consequently, nerve-sparing robot-assisted radical prostatectomy techniques were developed to prevent urinary incontinence and impaired erectile function. Many urologists have contributed to the development of techniques to preserve urinary continence and erectile function while achieving cancer control. However, the best surgical methods to achieve pentafecta after prostatectomy, including preservation of potency and continence, biochemical recurrence (BCR)-free survival, no postoperative complications, and negative surgical margins, have not been established^1^. Some of the previously reported methods require too many steps or a very detailed understanding of the anatomy.

Since 2015, we have developed a relatively simple and feasible approach that preserves structures of the pelvic floor muscles for nerve-sparing (NS) surgeries. We hypothesized that the use of an endopelvic fascia (EPF)-sparing technique would preserve as much of the surrounding tissue at the apex of the prostate as possible. In particular, structures that support the urethral and external urethral sphincter, such as the detrusor apron, pelvic fascia tendon arch, pubic-prostatic ligament, and prostatic venous plexus, should be preserved. The EPF on the NS side is not resected or split, and the prostate is dissected along the intrafascial plane, which is connected to the urethral side of the prostatic apex, to preserve the structures around the apex. The EPF on the non-NS side is resected. Herein, we present the detailed method for this EPF-preserving RARP. In addition, the functional outcomes and oncological outcomes of this method were assessed.

## Methods

### Study design and participants

We reviewed the medical records of all PCa patients who underwent RARP with EPF preservation by a single surgeon (K.M.) in Yokohama Rosai Hospital from January 2015 to March 2022. The criteria to perform NS surgery was based on a formula consisting of prostate-specific antigen (PSA) density, Gleason grade, percentage of positive cores, and the longest cancer length in the biopsy specimen. We have constructed a formula to calculate the possibilities of extra-prostatic extension (EPE) by univariate and multivariate analysis from data from 197 cases of prostatectomy in our hospital. ${\text{X}}= -7.461+0.01\times \left(\mathrm{PSA\, density }\left[{\text{ng}}/mL/{cm}^{3}\right]\right) + 0.499\times \left(\mathrm{Gleason\, Sum}\right)+0.015\times \left(\mathrm{percent\, positive \,core }\left[\%\right]\right)+0.315\times \left(\mathrm{longest\, cancer\, length }\left[mm\right]\right)$. ${\text{P}}\left(x\right)={{\text{e}}}^{X}/\left(1+{{\text{e}}}^{X}\right).\mathrm{ P}\left(x\right)$ is the percentage of the positive EPE. We calculate the P(x) for each side, and we set the cut-off as below 5% for indication of NS. Also, diagnosis using magnetic resonance imaging (MRI) and the patient’s opinion were considered when determining whether to perform NS surgery. This study was approved by the research ethics committee of Yokohama Rosai Hospital (Approval number 2023–11). The protocol for this research project conformed to the provisions of the Declaration of Helsinki. Informed consent was obtained from all participants or their legal guardians, with details about this study were disclosed on our institutional website and potential participants had the opportunity to decline enrollment in the study (opt-out).

### Data collection

The following variables were retrospectively extracted from medical records: age, body mass index (BMI), international prostate symptom score (IPSS), prostate volume, preoperative PSA level, clinical T stage, International Society for Urological Pathology (ISUP) biopsy grade, total number of biopsy cores, and number of positive biopsy cores. Surgical outcomes data were collected, including operative time, console time, and blood loss. Postoperative endpoints included pathological T stage, pathological N stage, surgical margins, complications, erectile function, and urinary continence. Lymph node (LN) dissection was performed mainly for patients with intermediate-risk until August 2017, but was omitted afterward since most of the patients eligible for NS-RARP are negative for LN metastasis. After discharge, patients were monitored every 3 months at an outpatient unit with medical interviews, physical examinations, and blood tests, including PSA. Urinary continence was defined as the use of 0 pads/day and potency was defined as possible intercourse or possible masturbation, based on interviews with the patient during the outpatient visit. For penile rehabilitation, 20 mg of sildenafil every two days was prescribed from postoperative day 3 for at least one month.

### Surgical procedure for EPF preservation

**Figure 1:**
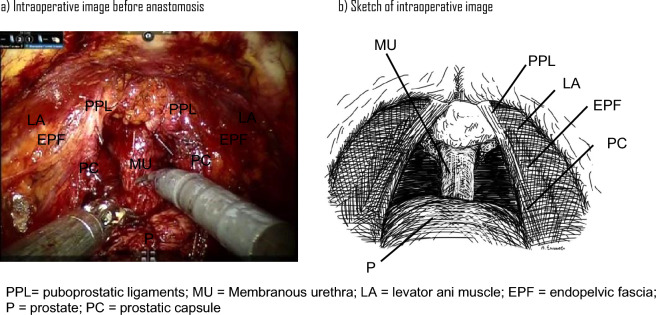
Intraoperative image of this procedure. (**a**) Intraoperative image before anastomosis. (**b**) Sketch of the intraoperative image. EPF—endopelvic fascia; LA—levator ani muscle; MU—membranous urethra; P—prostate; PC—prostatic capsule; PPL—puboprostatic ligaments.

All surgical procedures were performed using the da Vinci Si Surgical System (Intuitive Surgical, Sunnyvale, CA, USA). The patient was placed in a 30-degree head-down, supine position with the third robotic arm with six ports positioned on the patient’s left side. The camera port was set at 1.5 cm above the umbilicus. The pneumoperitoneum was administered at 12 mmHg. The peritoneum was incised widely on both sides along the lateral umbilical cord. Bilateral spermatic cords were cut, and the Retzius space was opened. The EPF on the NS side was not cut, but the EPF on the non-NS side was cut. A requisite minimum incision was made at the bladder neck, and the incision was advanced until the Foley catheter could be seen. The prostate was raised by moving the Foley catheter ventrally, and the retro-trigonal layer was exposed. The ductus deferens was detached and separated as much as possible. The seminal vesicle arteries were ligated using the Challenger® Ti-P (B. Braun, Melsungen, Germany) and cut. The exposed Denonvillier’s fascia was dissected to expose the prostatic capsule and veiled with intrafascial dissection. The communicating artery was cut between the prostatic capsule and the prostate after ligating with the Challenger® Ti-P. The prostatic capsule was spared from 1 o’clock to 11 o’clock, and then clipped and cut at 1 o’clock and 11 o’clock. For the left side, the Meryland bipolar forceps and the Prograsp forceps were switched to avoid conflict between the second and third arms. With a pneumoperitoneum of 15 mmHg, the dorsal vein complex (DVC) was cut athermally, and 3–0 Monocryl was used to close the DVC. After lowering the insufflation pressure to 12 mmHg, the prostate was dissected to the urethra (Fig. 1a,b). The urethra was cut cold, and the prostate was removed. Posterior wall augmentation was performed in two layers with 3–0 Monocryl. The first layer involved the connective tissue of the dorsal urethral mucosa and the Denonvillier’s fascia near the bladder. The second layer involved the connective tissue of the dorsal urethral mucosa and the retro-trigonal layer, which was close to the bladder neck. The vesicourethral anastomosis was performed using 3–0 Monocryl with a double-ended needle. A drainage tube was inserted into the Retzius space from the third port. The detailed surgical procedure is available in the supplementary video.

### Statistical analysis

Variables are expressed as means and standard deviation. Univariate Cox regression models were used to determine the effects of independent variables on BCR. A priori variables were selected based on previous literature. The final multivariable model was developed, including the variables that were statistically significant in the univariable analyses. Analyses of BCR-free survival and cancer-specific survival were performed using the Kaplan–Meier method. Two-sided *P* values < 0.05 were considered significant. Logistic regression analyses were performed using JMP Pro 16 (Statistical Analysis System Institute Inc, Cary, NC, USA), and other analyses were performed using GraphPad Prism version 10 for Windows (GraphPad Software, San Diego, CA, USA).

## Results

### Patient characteristics

Data from 100 patients who underwent NS-RARP with EPF preservation by a single surgeon at Yokohama Rosai Hospital were analyzed (Table 1). The mean age was 66 years, the mean BMI was 24, the mean IPSS was 9 points, the mean prostate volume was 45 ml assessed by MRI, the mean PSA was 9.2 ng/mL, and 99 cases were cT2 or less. The biopsy ISUP grade was 1 for 47 patients, 2 for 27 patients, 3 for 12 patients, and 4 for 14 patients. The mean number of positive biopsy cores was 2.7 out of 14. Table 2 summarizes surgical outcomes. The mean console time was 127 min, the mean blood loss was 95 ml. Forty-three patients underwent bilateral NS prostatectomies, and 57 patients underwent unilateral NS prostatectomies. The pathological T stages were pT0 in 1 patient, pT2a in 38 patients, pT2b in 12 patients, pT2c in 29 patients, pT3a in 13 patients, and pT3b in 7 patients. Limited lymph node dissections were performed in 24 patients, and all patients were negative for lymph node metastasis. The surgical margins were negative in 75 patients and positive in 25 patients.

**Table 1:** Patient characteristics.

Variables	Overall population (n = 100)
Age, years	66 ± 5.6
BMI, kg/m^2^	24 ± 2.4
IPSS	9 ± 7.3
Prostate volume, mL	45 ± 17
PSA, ng/mL	9.2 ± 7.9
Clinical T stage, n (%)	
cT1c	49 (49)
cT2a	32 (32)
cT2b	4 (4)
cT2c	14 (14)
cT3a	1 (1)
Biopsy ISUP Grade, n(%)	
1	47 (47)
2	27 (27)
3	12 (12)
4	14 (14)
Total number of biopsy cores, n	14 ± 4.6
Positive number of biopsy cores,n	2.7 ± 1.9

*BMI* body mass index; *IPSS* international prostate symptom score; *ISUP* international society of urological pathology; *PSA* prostate specific antigen.

Values are presented as mean ± standard deviation or number (%).

**Table 2:** Surgical outcomes.

Variables	Overall population (n = 100)
Console time, min	127 ± 30
Operation time, min	176 ± 35
Blood loss, ml	95 ± 114
Nerve sparing, n (%)	
Bilateral	43 (43)
Unilateral	57 (57)
LN dissection (limited, extended) (%)	24 (24, 0)
Pathological T stage, n (%)	
pT0	1 (1)
pT2a	38 (38)
pT2b	12 (12)
pT2c	28 (28)
pT3a	14 (14)
pT3b	7 (7)
Pathological N stage, n (%)	
pN0	24 (24)
pN1	0 (0)
pNx	76 (76)
Surgical margin, n (%)	
Negative	75 (75)
Positive	25 (25)
Positive surgical margin in pT2, n (%)	17 (22)
Positive surgical margin in pT3, n (%)	8 (38)
Follow-up period, years	4.2 ± 1.7

*LN* lymph node.

Values are presented as mean ± standard deviation or number (%).

### Complications

The complications for RARP with EPF preservation are shown in Table 3. Sixteen patients experienced 18 complications. One patient developed ureteral obstruction, which required ureteral stent insertion (Clavien-Dindo grade IIIa), and another patient developed ileus, which required nasogastric tube insertion (Clavien-Dindo grade II). Sixteen of 18 complications were classified as Clavien-Dindo grade I.

**Table 3:** Surgical complications.

Complications	Number (%)	Clavien-Dindo grade
None	83 (83)	NA
Ureteral stenosis	1 (1)	IIIa
Ileus	1 (1)	II
Inguinal Hernia	4 (4)	I
Incisional hernia	2 (2)	I
Lymphocele requiring longer drainage insertion	2 (2)	I
Intestinal damage	2 (2)	I
Wound infection	2 (2)	I
Anastomotic urine leakage	1 (1)	I
Acute urinary retentions	1 (1)	I
Postoperative bleeding	1 (1)	I
Hepatic impairment	1 (1)	I
Meatal stenosis	1 (1)	I

### Oncological outcome

**Figure 2:**
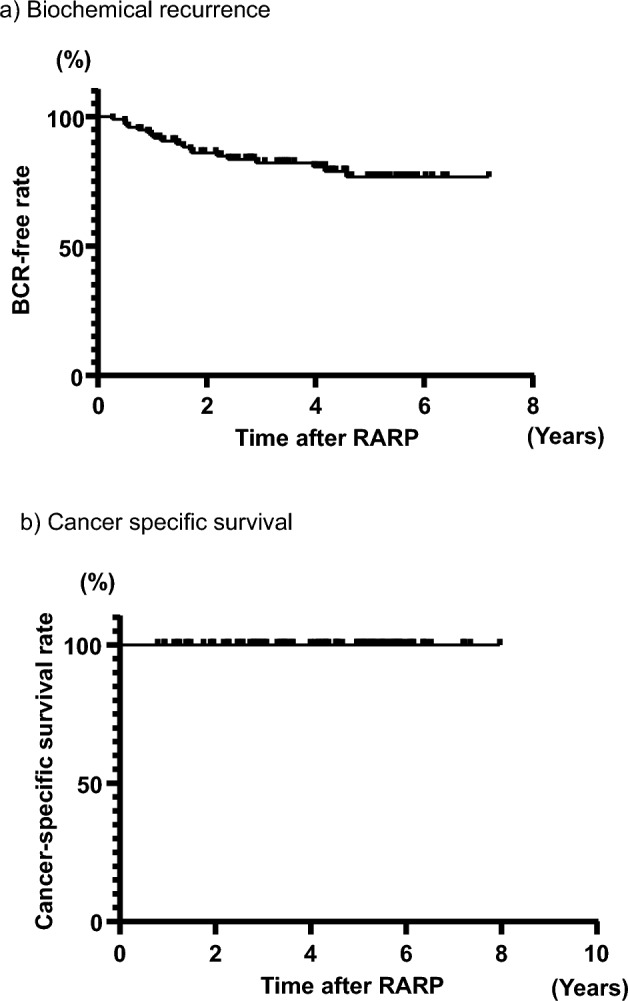
Oncological outcomes, including (**a**) BCR-free rate and (**b**) cancer-specific survival rate during the median observation period of 4.5 years.

During a mean follow-up period of 4.2 ± 1.7 years, 23 patients experienced BCR. The 1-year BCR-free rate was 92.7%, and the 5-year BCR-free rate was 76.7% (Fig. 2a). The effects of EPF-preserving NS surgeries on BCR were determined using the Cox-regression model. The following 5 factors were included in the model: PSA, NS (bilateral vs. unilateral), Gleason grade at RARP (≥ 4 vs. < 4), resection margin (positive vs. negative), and pT stage (≥ pT3a vs. < pT3a) (Table 4). The univariate analyses revealed that high PSA (*p* = 0.034), unilateral NS (p = 0.033), positive surgical margin (PSM) (*p* < 0.0001), and pT3a or more (*p* = 0.005) were significantly associated with BCR. The multivariate analysis using the significant factors from the univariate analyses showed that PSM was significantly associated with BCR (*p* = 0.0002). PSM were in the anterior area in 7 of 25 cases (28%) and in the lateral area in 7 of 25 cases (28%) (Supplementary Table 1). The cancer-specific survival (CSS) rate was 100% during the follow-up period (median, 4.5 years) (Fig. 2b).

**Table 4:** Factors associated with biochemical recurrence.

		Univariate analysis			Multivariate analysis		
		Odds ratio	95% CI	*P* value	Odds ratio	95% CI	*P* value
PSA		1.03	1.00–1.06	0.034	1.00	0.94–1.05	0.90
Nerve spare	Bilateral	0.38	0.15–0.98	0.033	0.46	0.17–1.25	0.13
	Unilateral	Ref					
Gleason grade at RARP	≥ 4	1.82	0.67–4.91	0.26			
	< 4	Ref					
Surgical margin	+	5.79	2.50–13.4	< 0.0001	5.67	2.25–14.3	0.0002
	−	ref					
Pathological T stage	≥ pT3a	3.26	1.42–7.44	0.005	10.1	0.39–262.2	0.16
	< pT3a	ref					

*BCR* biochemical recurrence; *PSA* prostate specific antigen; *RARP* robot-assisted radical prostatectomy.

### Functional outcome

**Figure 3:**
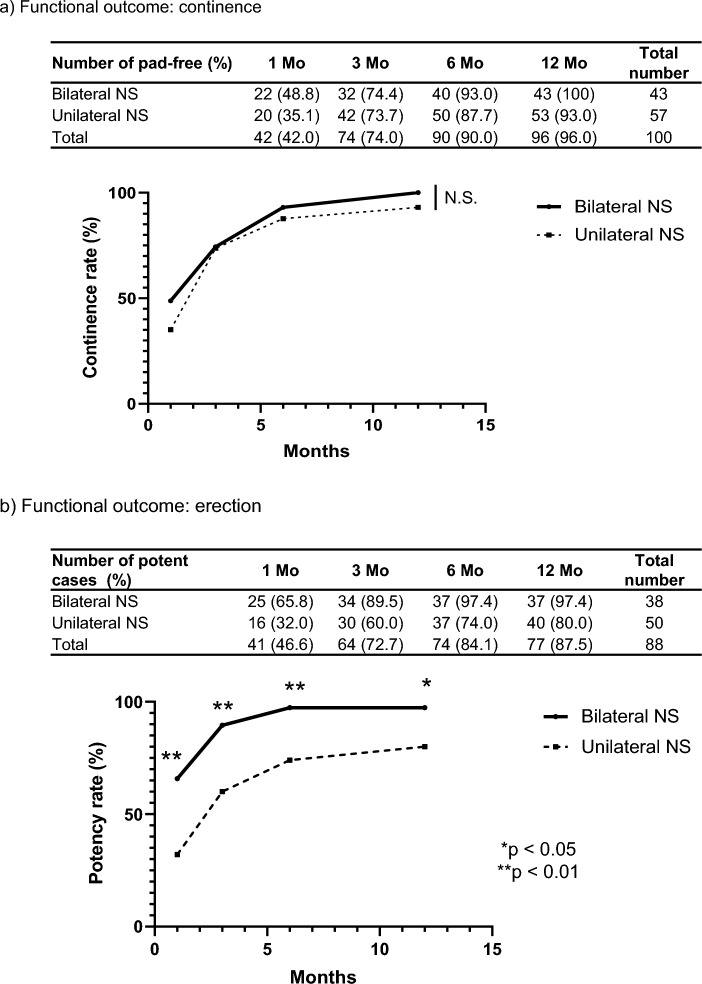
Functional outcomes after undergoing bilateral nerve-sparing or unilateral nerve-sparing endopelvic fascia-preservation surgery. (**a**) The percentages of patients who achieved complete no pad continence. (**b**) The percentage of patients who achieved satisfactory erectile function.

After the EPF-preservation surgery, 42% of patients at 1 month, 74% of patients at 3 months, 90% of patients at 6 months, and 96% of patients at 12 months achieved complete no pad urinary continence. Patients who underwent bilateral NS-RARP with EPF preservation tended to have better continence compared with patients who underwent unilateral NS-RARP, but the differences were not significant (bilateral NS, 1 Mo: 48.8%, 3 Mo: 74.4%, 6 Mo: 93.0%, and 12 Mo: 100%; unilateral NS, 1 Mo: 35.1%, 3 Mo: 73.7%, 6 Mo: 87.7%, and 12 Mo: 93.0%) (Fig. 3a).

88 patients with sexual function, including masturbation or intercourse before surgery were included in the analysis of the effects of EPF-preservation surgery on erectile function. After EPF-preservation surgery, 46.6% of patients at 1 month, 72.7% of patients at 3 months, 84.1% of patients at 6 months, and 87.5% of patients at 12 months achieved satisfactory erectile function. Potency rates were significantly better in patients who underwent bilateral NS prostatectomies compared with patients who underwent unilateral NS prostatectomies (1 month: 65.8% vs. 32.0%, *p* = 0.0024; 3 months: 89.5% vs. 60.0%, *p* = 0.0032; 6 months: 97.4% vs. 74.0%, *p* = 0.0028; and 12 months: 97.4% vs. 80.0%, *p* = 0.0204) (Fig. 3b).

## Discussion

Prostatectomy is the standard treatment for localized prostate cancer^2^. Although RARP is a less invasive procedure in terms of bleeding or operative time, postoperative urinary incontinence is a major complication, which markedly affects the quality of life of patients^3–5^. Although many surgical techniques have been developed to address the issue of urinary incontinence, no methodology to avoid urinary incontinence has been established^6–9^.

Wagaskar et al. reported that the Hood technique results in relatively good early postoperative urinary continence and low PSM rates^10^. The Retzius sparing approach also results in relatively good continence recovery^11,12^. However, these techniques are relatively new, and long-term BCR-free rates were not well reported. We developed a novel EPF-preserving technique, which we have employed since 2015. Thus, compared with other studies, the follow-up period for our study is longer, and oncological outcomes could be analyzed.

To compare functional, oncological, and surgical outcomes for our operative method with previously reported RARP procedures, we reviewed recent reports about RARPs, including conventional RARP with an anterior approach^10,12–16^ (Table 5). Although patient characteristics were different, console time, blood loss volume, and the rate of complications more than Clavien-Dindo grade III in our study were similar to previous studies. The overall PSM rate was 25% rate in our study. The PSM rate for pT2 tumors was 22% in our study, which was similar to PSM rates in previous RARP studies (6.5–32%)^17–22^. The reported PSM rates for the Retzius-sparing technique, another structure-preserving RARP, were 11–25% for pT2 tumors^11,23,24^, which were similar to the PSM rates in our study. The BCR-free rates in previous reports were also comparable to our BCR-free rates. Our cohort had a relatively better 1-year BCR-free rate of 93%. The use of NeuroSAFE may improve PSM rates and oncological outcomes, according to previous studies^10,16,25,26^.

**Table 5:** Comparison of patient characteristics and outcomes.

Study authors	Our series	Wagaskar et al. ^10^	Egan et al. ^12^	Lantz et al. ^13^	Hung et al. ^15^	Haese et al. ^16^
Study design	Retrospective	Prospective	Retrospective (Retzius sparing RARP vs. standard RARP)	Prospective (RARP vs. RRP)	Retrospective	Retrospective (RARP vs. RRP)
Number of cases to compare	100	300	70 (only Retzius sparing)	2,699 (only RARP)	111	3,783 (only RARP)
Method	EPF-preserving anterior	Hood	Retzius sparing	Anterior	Anterior	Anterior
Age (yr), median, mean	67, 66	64, NA	NA, 62	64, NA	NA, 65	64, 63
PSA (ng/mL), median, mean	6.7, 9.2	6, NA	NA, 7.2	6, NA	NA, 19	7.2, 9.2
Biopsy gleason group						
1 (%)	47	16	Mean Gleason group 2.6	51	Mean Gleason Sum 6.8	NA
2 (%)	27	40		34		
3 (%)	12	25		9.1		
4 (%)	14	13		5.7 (4 and 5)		
5 (%)	0	6				
Clinical T stage cT1 (%)	49	51	71	58	41	NA
cT2 (%)	50	35	19	37	51	
cT3 (%)	1	14	10	2.9	7.2	
Console time (min), median, mean	123, 127	118, NA	NA, 130	NA	NA	NA
Blood loss (ml), median, mean	50, 95	150, NA	100, NA	NA	NA, 221	200, 279
NS (bilateral, unilateral) (%)	100 (43, 57)	94 (80, 14)	84 (NA, NA)	84 (50, 34)	39 (20, 19)	98 (77, 21)
LN dissection (limited, extended) (%)	24 (24, 0)	NA	NA	12 (3.2, 8.5)	84 (NA/NA)	NA
Complications						
Grade I (%)	16	2.3	any 4.3	NA	5.4	NA
Grade II (%)	1	5.7			5.4	
Grade III (%)	1	1.7			1.8	
GradeIV, V (%)	0	0			0	
Pathological T stage						
pT0 (%)	1	0	0	0	0	0
pT2 (%)	78	81	67	72	41	73
pT3a (%)	14	19 (pT3a and pT3b)	20	27 (pT3a and pT3b)	57 (pT3a and pT3b)	19
pT3b (%)	7		13			8 (pT3b and pT4)
pT4 (%)	0	0	0	0.0	2	
PSM (%)	25	6 (NeuroSAFE)	24	22	50	12 (NeuroSAFE)
PSM in pT2 (%)	22	2.3	NA	NA	NA	7.8
PSM pT3 (%)	38	3.7	NA	NA	NA	17 for pT3a, 39 for pT3b/pT4
Follow-up period (years), median	4.5	NA	1	8	8.6	4
BCR-free rate (%)	1 year: 93 5 years: 77	NA	1 year: 87	8 years: 72	8.6 years: 63	89 (PS matched)
CSS rate (%)	100 at 4.5 years	NA	NA	99 at 8 years	97	NA
Definition of continence	Zero pad	Zero pad	Zero pad	Less than one pad/day	NA	0 or one safety pad
Continence (%)	1 month: 42	1 month: 83	12 month:73	8 years: 72		12 month: 90 (PS matched)
	6 months: 90	6 month: 94				
	12 months: 96	12 month: 95				
Definition of potency	Able to masturbate or intercourse	NA	Erection sufficient for sexual activity	IIEF-5 question 3: > = 2	NA	IIEF-5 question 2: > = 2
Potency (%)	1 month: 47		12 months: 66	8 years: 34		12 month: 83 (PS matched)
	3 months: 73					
	12 months: 88					

*BCR* biochemical recurrence; *CSS* cancer specific survival; *IIEF-5* international index of erectile function; *LN* lymph node; *NS* nerve spare; *PS* propensity score; *PSA* prostate specific antigen; *PSM* positive surgical margin; *RRP* radical retropubic prostatectomy; *RARP* robot-assisted radical prostatectomy.

The overall continence rate in our series (96% completely pad-free rate at 12 months after surgery) was better than the continence rates reported in previous studies. Furthermore, 100% of patients who underwent bilateral NS achieved zero-pad continence at 12 months. The potency rate of 88% at 12 months in this series was also better than the potency rates reported in previous reports, although the definition of potency is different among studies. Considering long-term cancer-specific survival after prostatectomy, improved functional outcomes are crucial for maintaining the quality of life for patients^3^. In contrast to previous studies, we reported longer oncological outcomes, as we began using this technique in 2015. CSS was 100% at the median follow-up of 4.5 years. Though androgen deprivation salvage therapy could decease the potency rate, the high continence rate with this EPF-preserving RARP is noteworthy.

Preservation of the pelvic floor anatomical structures is crucial to maintaining continence after RARP^8,10^. The EPF on the NS side is not resected in our approach, and the prostate is treated through the intrafascial dissection plane while maintaining a connection to the urethral side of the prostatic apex to preserve the structures around the apex. To achieve satisfactory erectile function, preserving the neurovascular bundle on the posterior side of the prostate and the anterior tissue around the prostate is necessary. This approach is in line with the basic functional ability for erections of complex neural organization around the prostate, including the anterior side of the prostate^27^. Despite not preserving the anterior tissue of the prostate from 11 o’clock to 1 o’clock, except for the Hood technique, we achieved high potency and continence rates of more than 90% at 12 months after surgery. Therefore, the tissue around 12 o’clock may not be essential for maintaining erectile function and continence after RARP. This technique can also be applied to patients with anterior side tumors, in contrast to the Hood technique. Furthermore, the anastomosis between the urethra and the bladder is easier to perform with a direct view of the urethra from the ventral side, as in our technique. Our approach is relatively simple; emphasizing EPF preservation may be the most important aspect of preserving pelvic floor anatomical structures. Our study is consistent with previous studies showing that preservation of pelvic floor tissue results in excellent functional outcomes^8–10,12,24,28^. Specifically, our technique is similar to the “Veil of Aphrodite” technique in that both techniques do not cut the EPF and enter the posterior side of the prostate to dissect the Denonvillier’s fascia to expose the prostatic capsule^28^. However, the biggest difference is that the “Veil of Aphrodite” technique starts their veil from inferolateral where the prostatic fascia reflects off the prostate, and coagulates or clips the prostatic pedicle, whereas we start dissection from 6 o’clock, and we cut the communicating artery between the prostatic capsule and the prostate one by one, after ligating with the Challenger® Ti-P. Also, the “Veil of Aphrodite” technique used cautery to cut the anterior side of the prostate and DVC together, but we spared the capsule from 1 o’clock to 11 o’clock, and then clipped and cut at 1 o’clock and 11 o’clock so that neural tissues around DVC can be preserved as much as possible].

This study has several limitations. First, this was a retrospective single-center study. In addition, this study included surgeries performed by a single surgeon. Another limitation is the selection of bilateral or unilateral NS, which may have affected the PSM rate. The decision to undergo bilateral or unilateral NS was ultimately determined by patients’ desires, although we recommended extracapsular extension of carcinoma based on several clinical parameters. Further multicenter randomized clinical trials are warranted.

## Conclusions

We developed a novel EPF-preserving NS-RARP with satisfactory functional outcomes and relatively long-term oncological outcomes. The present technique is simple and can be applied to patients who undergo NS-RARP.

### Supplementary Information

Supplementary Video 1.

Supplementary Information 1.

Supplementary Information 2.

## Data Availability

The datasets used and/or analyzed during the current study are available from the corresponding author upon reasonable request.
