# Supplementary material for: Novel nerve-sparing robot-assisted radical prostatectomy with endopelvic fascia preservation and long-term outcomes for a single surgeon
Source: Sci Rep. 2024 Jan 9;14:926. doi: 10.1038/s41598-024-51598-3 (PMC10776665; doi:10.1038/s41598-024-51598-3)
Supplement: Supplementary file 3 — Supplementary Information 2. [file 41598_2024_51598_MOESM3_ESM.docx]

**Supplementary information**

**Supplementary video. Surgical procedures for the EPF-preserving RARP with bilateral-NS**

A 62-year-old patient with a PSA of 4.1 ng/mL and 2 positive cores out of 12 with biopsy Gleason grade 1. The cancer was 1.5 mm/10 mm and 0.2 mm/18mm. The clinical stage was T1cN0M0. The operative time was 124 minutes, and the console time was 79 minutes. The pathological Gleason grade was 2 with 3 foci in the prostate. The patient was negative for extraprostatic extension and negative for surgical margins.

**Supplementary Table 1. The margin locations in patients with positive surgical margins.**
